# *In Vitro* Regeneration of Korarima (*Aframomum corrorima* (Braun) P. C. M. Jansen): A Threatened Spice and Medicinal Herb from Ethiopia

**DOI:** 10.1155/2022/8237723

**Published:** 2022-01-19

**Authors:** Tilahun Hailu, Teklehaimanot Haileselassie, Tileye Feyissa

**Affiliations:** ^1^Department of Applied Biology, Adama Science and Technology University, P. O. Box 1888, Adama, Ethiopia; ^2^Institute of Biotechnology, Addis Ababa University, P. O. Box 1176, Addis Ababa, Ethiopia; ^3^Department of Microbial,Cellular and Molecular Biology, College of Natural Sciences, Addis Ababa University, P. O. Box 1176, Addis Ababa, Ethiopia

## Abstract

Developing an *in vitro* regeneration system is very important to increase production and productivity of plants as well as for the conservation of rare and threatened medicinal plants like korarima (*Aframomum corrorima* (Braun) P. C. M. Jansen). To date, no study dealing with *in vitro* indirect regeneration system of korarima has been reported. Thus, in this study, we developed an efficient and reproducible protocol for *in vitro* regeneration of korarima via callus. The procedure involved soaking seeds in 50% H_2_SO_4_ for 16 h that resulted in 92.5% germination on plant growth regulators (PGRs)-free half-strength Murashige and Skoog (MS) basal medium after a month. Shoot and rhizome induction rate of 93.75% was obtained on the MS medium containing 1.5 mg/l BAP in combination with 0.1 mg/l IBA after five weeks. Whitish yellow friable callus was obtained from rhizome culture taken from *in vitro* grown plantlets. The MS medium containing 2.0 mg/l 2, 4D in combination with 0.5 mg/l kinetin, resulted in 77.5% callus induction. The shoot regeneration rate of 45% was obtained from callus on the MS medium containing 2.0 mg/l TDZ in combination with 0.5 mg/l IBA. The mean shoot number of 10.83 per explant was obtained upon multiplication on the MS medium containing 1.5 mg/l BAP with a mean shoot height of 5.37 cm. The best rooting responses were obtained on half MS medium supplemented with 0.5 mg/l IAA resulting in a mean number of root of 18.59, mean root length of 9.71 cm, and mean shoot height of 7.32 cm. The plantlets showed 75% survival efficiency after acclimatization. The present regeneration protocol offers a conceivable system towards effective conservation and genetic improvement of the crop by increasing the efficiency of genetic transformation.

## 1. Introduction

Korarima (*Aframomum corrorima* (Braun) P. C. M. Jansen) (Zingiberaceae) is a threatened plant species native to Ethiopia [1, 2]. The plant is a perennial herb that suitably grows up to two meters high in shady habitats. Salient features of the plant include the possession of subterranean rhizome with an aerial leafy shoot often covered by sheathing leaf bases and its inflorescence and fruits that arise from the rhizome near the base of a leafy stem. The plant has a relatively broader adaptation and better tolerance to moisture stress [3].

Korarima occurs as a cultivated crop only in Ethiopia where its initial domestication is assumed to take place as evidenced by the co-occurrence of both wild and cultivated genotypes [1, 4]. Although it is used in nationwide basis, its cultivation is most seen in forested areas of the southern and southwestern parts of the country where suitable shade trees and favorable warm-humid climate with humus-rich soils are found [5].

Korarima is either cultivated in home garden or directly collected from wild in the natural forest or managed forests as in Sheka, Bench Sheko, and Kafa zones. The plant is propagated both sexually by seed and vegetatively by rhizome. After planting, it requires three to five years for maturity and was harvested from August to November all the year round for more than seven years of economic life [6]. Dried capsules holding the seeds are the valuable and harvestable part of the plant as it owns wide range of flavor and aroma of high economic, pharmaceutical, and industrial importance [4, 7]. The crop is also important for soil conservation as the rhizomes and leaves spread on the ground covering and protecting the soil from erosion in hilly areas [8].

In Ethiopia, korarima seeds are considered the leading spice for which it is ground and usually mixed with other spices to flavor all kinds of sauces. It is also used to flavor coffee, tea, bread, and butter [9, 10]. The plant is also used in traditional medicine across the country as a carminative, purgative, and tonic agent for treating ailments of humans and domestic animals [11]. Essential oil in seeds and fruits' husk of korarima contains a significant amount of natural monoterpenes and their derivatives known for their diverse pharmaceutical properties [7, 12]. It has a good economic importance to the local growers and the country as its seeds, either in dried forms or as oleoresin and essential oils extract, reach high prices in local, national, and international markets [13, 14].

Irrespective of its immense importance, there are major production bottlenecks such as lack of a steady supply of high yielding improved varieties with improved agronomic practices like propagation methods and plant disease. In this regard, the conventional propagation of korarima by seeds is time-consuming and laborious requiring maximum care while preparing the seeds [15]. The slow seed germination and subsequent seedling establishment were concerns of korarima-producing farmers [8, 16]. Dormancy associated with the hard seed coat and low food reserve in the seed endosperm was the major reason for the very slow growth of the seedlings [17, 18]. Although korarima is mainly propagated by the vegetative method using rhizomes as planting materials, the need for a large amount of rhizomes and slow multiplication rate of rhizomes are also critical problems [16, 19, 20]. The destructive harvesting of the rhizomes for vegetative propagation leads to the possibility of losing the mother plant [21].

Moreover, the extent of korarima genetic resource is dwindling as its natural habitat in the southern and southwestern humid afro-montane forests is being diminished due to agricultural expansion and logging in part of its shade trees. Status of the plant species was reported to be declining outside protected areas [8, 22]. Thus, collecting germplasms from different geographic locations and safeguarding them at *in situ* and *ex situ* sites are important for conservation and sustainable utilization of the plant.

*In vitro* regeneration protocol is a prerequisite for the conservation of rare-threatened plant species, crop improvement through genetic transformation, and facilitating easy exchange of germplasm. Indirect regeneration culture offers opportunities to produce “new” korarima genotypes resulting from mutagenesis and somaclonal variation, as well as transgenes [23, 24]. To date, very few tissue culture studies dealing with micropropagation or direct organogenesis of korarima by using lateral buds from rhizomes [18] and using shoot tips [16, 25] have been reported, and no study about *in vitro* regeneration system *via* callus has been reported.

Therefore, this research work was initiated to develop an efficient and reproducible protocol that enables an *in vitro* regeneration of korarima using callus tissue produced from aseptic rhizome explants.

## 2. Materials and Methods

### 2.1. Seed Germination and Shoot Induction

Seeds of fresh ripe fruits were obtained from the Jimma Agricultural Research Center (JARC), Ethiopia, and stored under dry conditions at room temperature until the experiments were started. Voucher specimens were collected, pressed, and deposited in the National Herbarium of Addis Ababa University (AAU). The plant identification was performed both in the field and at the National Herbarium of AAU. Furthermore, the plant name was checked and verified from the African plant database https://africanplantdatabase [26] and https://www.worldfloraonline.org/ [27] for its nobility.

The experiment used the MS [28] basal media supplemented with 30 g/l sucrose. Cytokinins such as BAP, TDZ, and kinetin (KN), as well as the auxins such as IAA, IBA, and NAA, were used for different experiments. The pH of the media was adjusted to 5.7 before 7 g/l agar was added as a solidifying agent. Beforehand, the medium was autoclaved at 105 kPa and 121°C for 20 minutes. The autoclaved medium was allowed to cool in a sterile environment after which it was ready for use or retained in the media room for a maximum of three days prior to use. Cultures were maintained in a relatively controlled growth room at a temperature of 27°C ± 2°C and a light intensity of 1000–2000 lux from cool white fluorescent lamps under 16 h photoperiod.

For the initiation of aseptic culture, capsules were cut open to release seeds, dried in shade for three days, and kept in sterile plastic bags. The seeds were taken out of plastic bags using forceps and washed three times with tap water and commercial liquid detergent. The washed seeds were kept under running tap water for 10 minutes. The seeds were then transferred to the laminar flow hood, washed in 70% (v/v) ethanol for one minute, and rinsed three times in sterile distilled water. This was followed by surface sterilization with sodium hypochlorite (NaOCl) solution that contains 5% active chlorine, at 20% NaOCl (1% active chlorine) concentrations for 20 minutes of exposure time. The seeds were rinsed five times with sterile distilled water followed by culturing in glass jars containing 50 ml plant growth regulator (PGR)-free half-strength MS basal solid medium. For optimum *in vitro* seed germination, to overcome the hard seed coat, seeds were soaked in 50% H_2_SO_4_ solution for 16 hours before sowing following [8, 25].

For shoot induction, shoot apices (1–1.5 cm) were taken from 45 days old *in vitro* germinated seedlings and cultured on agar (0.7% plant propagation agar)-solidified MS basal media supplemented with 0.5, 1, 1.5, 2, 2.5, and 3 mg/l BAP alone and each BAP level in combination with 0.1 and 0.2 mg/l IBA. The liquid MS medium fortified with the above six levels of BAP was also used in the experiment. Therefore, the experiment was laid out using a complete random design (CRD) with a treatment combination of six concentrations of BAP and two concentrations of IBA that provides 1 × 6 factorial combinations in both cases of solid and liquid media and 6 × 2 for solid media.

Subculturing was carried out twice on the same fresh medium and maintained in growth room until suitable rhizomes develop for use as explant in subsequent callus induction experiment. The percentage of shoot tips that developed rhizomatous shoots, the number of shoots per explant, the height of microshoot, and number of leaves per microshoot were recorded after six weeks.

### 2.2. *In Vitro* Regeneration of Korarima

Young rhizomes (0.5–1.0 cm long) induced on *in vitro* shoot tip cultures were cut into small pieces and punched using forceps, wounded and cultured on the MS medium containing 30 g/l sucrose and 2, 4-D (1.0, 2.0, 3.0, 4.0, and 5.0 mg/l), NAA (1.0, 2.0 and 3.0 mg/l) and 2, 4-D (1.0, 2.0, 3.0, and 4.0 mg/l) in combination with KN (0.5 and 1.0 mg/l) and NAA (0.5 and 1.0 mg/l) each. Accordingly, the experiment was laid out using the CRD with 5 × 3 and 4 × 2 × 2 factorial combinations. Five pieces of rhizomes were cultured in Magenta culture vessels. The culture was transferred to the same fresh medium every four weeks and maintained in darkness for 12weeks at room temperature. The percentage of rhizomes that induced callus with their color and texture was observed and recorded after nine weeks of the initial culture.

After three months, the produced calli were transferred to the shoot regeneration medium containing 0.5, 1.0, 1.5, 2.0, and 3.0 mg/l BAP and TDZ alone as well as 1.0, 2.0, and 3.0 mg/l BAP and TDZ in combination with 0.5 mg/l and 1.0 mg/l IBA each. Therefore, the experiment was laid out in the CRD with 5 × 5 and 3 × 3 × 2 factorial combinations. Then, the cultures were kept for a 12-hour photoperiod and covered with loose transparent soft papers for fifteen days. The cultures were then uncovered and maintained under eight-hour light conditions at 25 ± 2°C for another 15 days after which they were transferred to 16-hour photoperiod. Regenerated shoots were then separated and transferred on the same fresh medium. The percentage of calli that regenerated shoot, the number of shoots proliferated per explant, and shoot height were recorded after nine weeks.

Microshoots that responded well were transferred to a shoot multiplication medium. The shoot multiplication medium was the MS medium containing BAP, TDZ, and KN at 0.5, 1.0, 1.5, 2.0 ,and 2.5 mg/l each and 1.0, 2.0, and 3.0 mg/l BAP combined with 0.1, 0.3, and 0.5 mg/l IBA. Thus, the experiment was arranged in a 3 × 5 and 3 × 3 factorial combinations in CRD. After five weeks, the multiplied shoots were separated and subcultured on the same fresh medium for three weeks. The number of shoots proliferated per shoot bud/microshoot explant and the length of shoots were recorded after six weeks of initial culture. However, the number of shoots and length of shoots produced after initial culture as well as at two successive subcultures within three weeks were recorded, and the mean values were considered for analysis.

For rooting, microcuttings (2–3 cm long) produced through regeneration were excised and cultured on the half-strength MS medium supplemented with each of IBA, IAA, and NAA at 0.25, 0.5, 0.75, 1.0, 1.25, and 1.5 mg/l concentrations using the CRD of 3 × 6 factorial combinations. The number of roots, root length, and shoot height were recorded after five weeks of culture.

After five weeks, well-rooted plantlets were removed from the culture vessels, and the roots were washed carefully under slightly warm water to facilitate the removal of adhering agar from the root surface, rinsed in a fungicidal solution of 3% Kocide‐101, and transferred to a sterilized potting mix of forest soil, compost, and sand at a ratio (v/v) of 1 : 2 : 1, respectively. The plantlets were then kept under a plastic cover of high humidity (80–90%) to prevent desiccation for ten days. Starting from the seventh day, the relative humidity within the plastic cover was reduced gradually to reach about 60% at the end of the tenth day. After the tenth day, the plantlets were transferred to a 70% shade net, where they were retained for a month. Later, they were transferred to a 30% shade net and maintained for another month, prior to transfer to the field. The percentage of survived plants was recorded after two months in greenhouse.

### 2.3. Experimental Design and Data Analysis

All experiments were laid in the CRD with factorial treatment combinations, having five explants per culture vessel and four replications per treatment. All the experiments were repeated two times to ensure reproducibility of the results and the average were considered for analysis. Control experiment was laid together in all cases.

Means of the data from the two repetitions for each experiment were subjected to statistical analyses using SAS [29] statistical software version 9.2, and ANOVA was constructed, followed by mean separation using appropriate procedures (REGWQ). When the ANOVA indicated significant treatment effects (5%, 1%, or 0.1%) based on the F-test, a probability level of 0.05 was used to determine which treatments were statistically different from the other.

## 3. Results and Discussion

### 3.1. Seed Germination and Shoot Induction

#### 3.1.1. *In Vitro* Seed Germination

**Figure 1:**
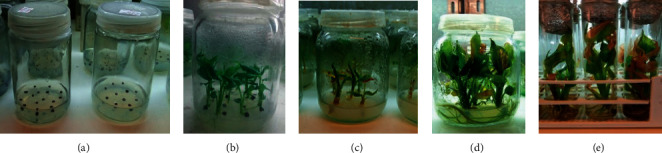
Seed germination and shoot induction: (a) korarima seeds cultured on 0.7% agar-solidified half-strength MS medium sterilized with 20% NaOCl solution for 20 min; (b) five-weeks-old seedlings developed from seeds cultured on solid medium treated with 50% H_2_SO_4_ solution for 16 hours; (c) shoot tip culture; (d) shoots initiated on MS medium containing 1.5 mg/l BAP combined with 0.1 mg/l IBA after five weeks; (e) shoots initiated on liquid MS medium containing 1.5 mg/l BAP combined with 0.1 mg/l IBA after five weeks. Bars = 1 cm.

Following the method explained under 2.1, after five weeks of *in vitro* growth and development on the PGR-free half-strength MS solid media, germination percentage of 92.5% was obtained from one-week-old seeds soaked in 50% H_2_SO_4_ for 16 hours (Figures 1(a) and 1(b)).

The result indicated that korarima seeds show dormancy or weak germination and poor seedling development that can be released by acid pretreatment for a certain period. Several other studies [30–33] indicated that concentrated sulfuric acid treatments are effective in breaking hard seed coat. The influence of sulfuric acid in promoting seed germination might be due to the highly desiccant property of the acid on the seed coat, thereby letting easier imbibition and oxygen diffusion [9, 32]. On the other hand, impeding effects of monoterpene hydrocarbons on germination and growth of seedling of different plant species were reported [34]. In this regard, it has been reported that monoterpene compounds were highly dominant in korarima seed essential oil [7, 35]. Soaking of korarima seeds in 50% H_2_SO_4_ for 12–24 hours, reported by [25], was also found to be effective in breaking the dormancy and stimulating germination.

#### 3.1.2. Shoot Induction

The ANOVA revealed that BAP alone as well as combined with IBA had a very highly significant (*p* < 0.0001) effect on the rate of shoot induction, number of shoot, shoot length, and number of leaf per shoot (Table 1). Accordingly, the highest rate of shoot induction (93.75%) was obtained on the MS medium containing 1.5 mg/l BAP combined with 0.1 mg/l IBA (Figure 1(d); Table 2) with good mean number of shoots per explant (3.33), mean shoot length (4.35 cm), and leaf per shoot (8.85). The second best shoot induction rate (91.25%) was attained on both 1.5 mg/l BAP and 1.5 mg/l BAP combined with 0.2 mg/l IBA. Shoots initiated on liquid MS supplemented with different PGR levels were taller and with more leaves but lesser in the number of shoots compared to similar treatments of solid media (Figure 1(e)).

Shoot bud induction ability of korarima shoot tip explants was enhanced with an increase in the concentration of BAP from zero to 1.5 mg/l and declined with further addition of BAP (Table 2). Such effects might be caused due to the exogenous application of cytokinins, which release the shoot buds from apical dominance. However, addition above the certain optimum limit reduced the shoot induction rate by inhibiting the availability of endogenous auxin needed to promote shoot bud formation usually interacting with cytokinins.

Application of exogenous auxin does not promote axillary shoot proliferation. Nevertheless, low concentration of auxin, together with high level of cytokinin, is often useful for inducing shoot bud [36, 37]. This effect was seen in the present study that the synergy of BAP and IBA produced the best response when a relatively low concentration (0.1 mg/l) of IBA is combined with relatively high level (>1 mg/l) of BAP than both increased or both decreased (Table 2). The combination of 0.5 mg/l BAP and 0.2 mg/l IBA produced a weak response (41.25%) of shoot bud induction rather than callus formation, whereas the same level of BAP combined with 0.1 mg/l IBA gave slightly a better response (55%) of shoot bud formation. Increasing the level of BAP to 2.5 mg/l securing the 0.1 mg/l IBA, however, had shown more improvement (77.5%) in shoot induction as discussed above. This supports the fact that it is the ratio of auxin to cytokinin, not the absolute level of auxin that suppresses shoot bud development and growth [37].

The present result is comparable to the report by [25] regarding shoot induction response of korarima shoot tip explants on MS medium fortified with 1 mg/l BAP. It also partially agrees with [37] who reported 90% shoot bud induction rate and mean values of 1.4 shoot per shoot tip explant, shoot length of 2.27 cm, and 2.17 leaves per shoot as the highest records from shoot tip explants of korarima on MS medium containing 1 mg/l BAP together with 0.1 mg/l NAA.

### 3.2. *In Vitro* Regeneration of Korarima

#### 3.2.1. Callus Induction

**Figure 2:**
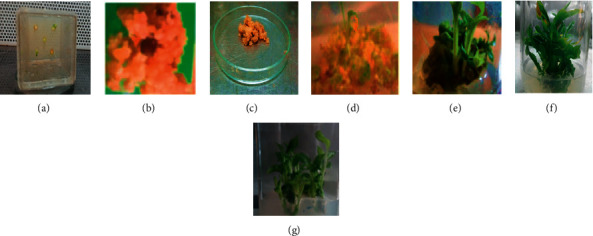
*In vitro* regeneration of korarima: (a) rhizomes cultured on callus induction media; (b) friable callus produced on the MS medium containing 3.0 mg/l 2,4-D combined with 0.5 mg/l KN after 10 weeks; (c) friable callus ready for shoot regeneration; (d) shoots developed from callus on shoot induction MS medium containing 2.0 mg/l TDZ combined with 0.5 mg/l IBA after two weeks; (e) shoots regenerated on 2.0 mg/l TDZ combined with 0.5 mg/l IBA after four weeks; (f) shoots proliferated after the transfer of shoot tips on 2.0 mg/l TDZ combined with 0.5 mg/l IBA; (g) shoots proliferated after the transfer of shoot tips on 2.0 mg/l BAP combined with 0.5 mg/l IBA. Bars = 1 cm.

The rhizome explants started showing signs of callus formation after six weeks on callus induction media. After ten weeks, 66.67% of the treatments induced callus and a range of color and texture variation was observed among different PGR treatments. The ANOVA revealed that the concentration of 2, 4-D, KN, and NAA had a very highly significant effect (*p* < 0.0001) on the rate of callus induction (Table 3). MS basal media containing 2.0 mg/l 2, 4-D combined with 0.5 mg/l KN resulted in 77.5% callus induction producing whitish yellow and friable textured callus (Figure 2(b)). The medium supplemented with 3.0 mg/l KN combined with 0.5 mg/l NAA produced no callus.

Callus induction was directly related to the concentration of growth regulators [36]. Auxins such as 2, 4-D and NAA promote callus induction at moderately high concentration. However, supra-optimal concentrations are toxic to the explants [23]. Inline to this, the present study revealed that the callus induction rate was increased with an increase in the concentration of 2, 4-D and NAA from zero up to 2.0 mg/l and went on decreasing above that level (Table 4). On the other hand, high-concentration (3.0 mg/l) KN combined with 1.0 mg/l NAA promoted adventitious shoot formation rather than callus formation.

#### 3.2.2. Shoot Regeneration

The concentrations of BAP alone, TDZ alone, BAP combined with IBA, and TDZ combined with IBA were found to be very highly significant (*p* < 0.0001) on the rate of shoot regeneration, the number of shoots produced, and the height of shoots obtained as evidenced by ANOVA (Table 5).

The highest frequency of shoot regeneration (45%) was obtained on the MS medium containing 2.0 mg/l TDZ combined with 0.5 mg/l IBA followed by 43.33% obtained on the medium fortified with 2.0 mg/l BAP combined with 0.5 mg/l IBA (Table 6). After nine weeks on culture, the mean numbers of 4.45 and 4.34 shoots were produced on the above two media compositions, which was almost the same (Figures 2(f) and 2(g)).

It is well known that PGR composition influences the direction of growth and development of cultured dedifferentiated calli [16, 38]. Hence, the rate of *in vitro* shoot regeneration could be affected by PGR composition of the growth medium.

The synergy of TDZ and IBA was found to be effective for the shoot regeneration of korarima. The two highest regeneration rates (45% and 43%) were obtained on the MS medium containing 2.0 mg/l TDZ combined with 0.5 mg/l IBA and MS medium fortified with 2.0 mg/l BAP combined with 0.5 mg/l IBA (Table 6). The mean numbers of shoots produced on the above two media compositions after nine weeks were almost the same (4.45 and 4.34), whereas the mean height of shoot varied from 1.80 on the former and 2.4 cm on the latter.

Callus tissues cultured on the PGR-free MS medium, MS medium fortified with 3.0 mg/l BAP, and MS medium fortified with 3.0 mg/l TDZ did not show shoot regeneration. This result backs the actual knowledge about the need for the interactive activity of cytokinins and auxins in promoting cell division, growth, and development in plants [17, 36, 37].

#### 3.2.3. Shoot Multiplication

The ANOVA revealed that the level of BAP, TDZ, and KN, as well as BAP combined with IBA had a very highly significant effect (*p* < 0.0001) on *in vitro* shoot multiplication rate and shoot height of korarima (Table 7).

**Figure 3:**
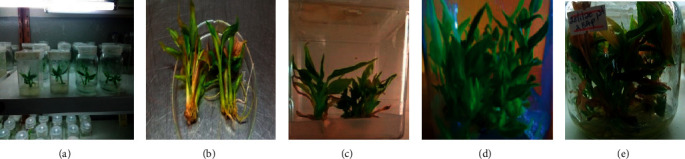
*In vitro* shoot multiplication of korarima: (a) microshoots cultured on shoot multiplication medium; (b) shoots proliferated on the MS medium containing 1.5 mg/l BAP after four weeks; (c) shoots proliferated on the MS medium containing 2.0 mg/l BAP after four weeks; (d) shoots proliferated on the MS medium containing 1.5 mg/l BAP after the second subculture, that is, 9 weeks; (e) shoots proliferated on the MS medium containing 2 mg/l BAP after the second subculture, that is, 9 weeks. Bars = 1 cm.

The highest mean number of shoot per explant (10.83), with a mean shoot height of 5.37 cm, was obtained on the MS basal medium supplemented with 1.5 mg/l BAP (Figures 3(b) and 3(d)) followed by the medium containing 2.0 mg/l KN that proliferated 9.66 shoots per explant with a mean shoot height of 4.72 cm. The medium containing 1.0 mg/l TDZ produced a mean shoot number of 9.25 with a mean shoot height of 5.21 cm. The synergetic effect of 2.0 mg/l BAP combined with 0.1 mg/l IBA resulted in a mean shoot number per explant of 9.24 and a mean shoot height of 5.29 cm (Table 8). In all cases, shoots cultured on the medium containing 2.0 mg/l and above cytokinin showed stunted growth, curled leaves, and weak shoot buds.

*In vitro* propagation is an advanced technique for producing a large number of genetically uniform and pathogen free plants in limited time and space [39], which is based on the successful adjustment of the type and combinations of plant growth regulators. *In vitro* shoot multiplication implies multiplication from one culture to many cultures and then many cultures to further many cultures, which is essentially a major criterion for successful commercial micropropagation. In the present study, the use of different types and concentrations of plant growth regulators highly affected the *in vitro* proliferation rate of korarima and subculturing was also found to be important for obtaining the optimum shoot multiplication result.

An increase in the number of shoots per shoot bud culture with an increased level of BAP and KN from zero to 1.5 or 2.0 mg/l (Table 8) is due to the effect of cytokinins in releasing lateral buds from dormancy or breaking apical dominance by inhibiting the level of endophytic auxins [37, 40]. However, too high concentration of cytokinin causes the production of many small shoots, which typically fail to elongate, and it also causes the leaves to have an unusual shape [36]. This was manifested in the present study that shoots cultured on the medium containing more than 2.0 mg/l cytokinin showed stunted growth, curled leaves, and weak shoot buds.

The proliferation rate had shown a progressive increase from the initial culture to the first subculture and second subculture by an average of 16.66% and 25%, respectively. Subculturing by trimming the base promotes the shoot proliferation rate as it is easier to establish juvenile than adult explants *in vitro*. Adult propagules respond poorly in micropropagation due to phenolic exudates, which causes blackening [38, 41]. Additional reason for subculture is that the growth of plant in a closed culture vessel in time leads to the buildup of toxic metabolites and the exhaustion of the medium [36]. In this study, yet further steps of subcultures that enable the evaluation of the overall progress up to the declining stage were not carried out.

Most of the shoot buds cultured on the MS medium containing 1.0 mg/l BAP combined with 0.5 mg/l IBA resulted in callus formation, whereas those at 2.0 mg/l and 3.0 mg/l BAP combined with the same 0.5 mg/l IBA resulted in shoot formation. This effect might be attributed to unsuitable proportion among cytokinin and auxin for proper stimulation of shoot growth [24].

In the present study, the MS medium containing 1.5 mg/l BAP resulted in the highest number of shoots per explant. The mean number of shoots reported by [16] on this medium was only 3.0, whereas it was 10.83 in the present study. On the other hand, [25] reported the mean shoot number of 10.33 shoots with 2.08 cm mean height per shoot bud on the MS medium containing 6.0 mg/l BAP, which is similar to the present study with respect to mean number of shoot obtained but different in the BAP level. Such variations might happen due to factors such as genotype difference and explants used.

Mean shoot number of 16.6 per explant has been reported by [18] on the MS medium supplemented with 0.5 mg/l TDZ, which is in agreement with the present study. In relation to this, [41] had indicated the efficiency of TDZ in promoting shoot proliferation through the induction of axillary bud release and growth of adventitious buds at relatively lower concentrations than the adenine-type cytokinins, for example, BAP. According to [42], these effects were mainly attributed to the stimulation of synthesis or accumulation of endogenous cytokinins by TDZ. Factors such as an increase in synthesis, a decrease in catabolism, or release of biologically active cytokinin molecules from nonactive storage forms by the activity of TDZ could be additional reasons [43].

#### 3.2.4. *In Vitro* Rooting and Acclimatization

All (100%) of the microcuttings cultured on the half-strength MS medium containing 0.5, 1.0, and 2.0 mg/l of the above auxins were rooted after four weeks. The ANOVA also indicated that the variations in the concentration of the auxins had a very highly significant (*p* < 0.0001) effect on the mean root number and length as well as shoot height (Table 9).

**Figure 4:**
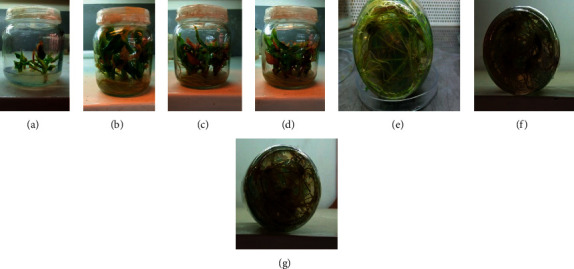
*In vitro* elongation and rooting of *A. corrorima*: (a) shoots cultured on rooting medium; (b) elongated plantlets on the 1/2 MS medium containing 0.75 mg/l IBA after five weeks; (c) elongated plantlets on the 1/2 MS medium containing 0.5 mg/l IAA after five weeks; (d) elongated plantlets on the 1/2 MS medium containing 0.75 mg/l NAA after five weeks; (e) roots developed on the 1/2 MS medium containing 0.75 mg/l IBA; (f) roots developed on the 1/2 MS medium containing 0.5 mg/l IAA; (g) roots developed on the 1/2 MS medium containing 0.75 mg/l NAA. Bars = 1 cm.

The highest mean number of roots (18.59), mean root length (9.71 cm), and mean shoot height (7.32 cm) were recorded from the half-strength MS medium containing 0.5 mg/l IAA followed by the half-strength MS medium containing 0.75 mg/l IBA resulting in a mean root number of 16.59, mean root length of 8.16 cm, and mean shoot height of 7.6 cm (Figure 4). The half-strength MS basal medium containing 0.75 mg/l NAA also showed a comparable result (Table 10). The roots formed in the half-strength MS medium were normal, long, and thick with many thin branches as compared to those obtained on the full-strength MS medium that was carried out as a preliminary experiment in the present study.

Rooting is an important process to the success of micropropagation. Without an effective root system, plant acclimatization is difficult and the rate of plant propagation may be affected substantially [44]. Concentrations of auxins, culture conditions, and medium strength are factors supposed to influence *in vitro* rooting. In the present study, all IAA, IBA, and NAA were found to be effective in promoting root formation and enhancing shoot elongation on the half-strength MS basal medium.

Similar to the present result, [8] reported 75.5% root formation of korarima displaying a mean root number of 13.67 with a root length of 0.72 cm on 1.0 mg/l IBA. [17], on the other hand, reported the formation of fine and healthy roots on the PGR-free MS medium. In the present study, however, shoots cultured on the PGR-free MS medium as a control produced a mean root number of 5.64 having a mean length of 2.89 cm and mean shoot height of 2.36 cm showing that better root number with good appearance and shoot height could be obtained on medium containing moderate (0.5 mg/l–1.0 mg/l) auxins than the PGR-free medium (Figures 4(b)–4(g)). The rooting response was decreased with a further increase in auxin beyond 1.0 mg/l. The inhibition of root growth and development might partly be due to ethylene release triggered by high auxin concentration [24, 37, 45].

Parallel to [16, 23], the number of roots induced per shoot increased under moderate concentrations of auxin, and when the concentrations were above the certain optimal level, callus formation was observed to be promoted in this study, supporting the general knowledge established about PGR use in plant tissue culture [36].

**Figure 5:**
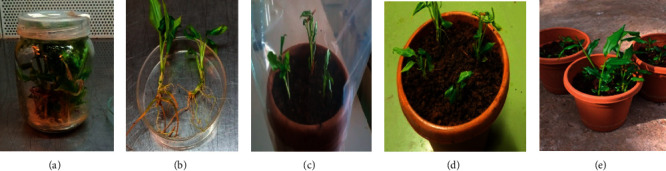
Acclimatization of *in vitro* grown plantlets of *A. corrorima*: (a, b) plantlets ready for acclimatization; (c) plantlets in the pot of sterilized forest soil: compost: sand (1 : 2 : 1) covered with plastic; (d) plantlets adapted to the external environment after 10 days of plastic cover; (e) acclimatized plants after two months. Bars = 1 cm.

After rooting, 143 plantlets were transferred to pots (3 plantlets per pot), containing a mixture of forest soil, compost, and sand in 1 : 2 : 1 ratio. The pots were then shielded with polyethylene plastic covers and placed in the greenhouse for acclimatization to outer environmental conditions, out of which, 107, that is, 75%, were survived and established well in pots after two months (Figure 5).

## 4. Conclusions

An *in vitro* regeneration protocol for korarima was established. The protocol could provide a possible system towards effective germplasm conservation and genetic improvement of the crop, and increase the efficiency of transformation protocols employing efficient regeneration system.

## Figures and Tables

**Table 1:** ANOVA summary of effect of BAP and BAP*∗*IBA on *in vitro* shoot induction of *A. corrorima*.

Source	DF	Mean square	*F* value	Pr > *F*
BAP	6	1723.57456^*∗∗∗*^	139.11	<0.0001
BAP*∗*IBA	11	394.79167^*∗∗∗*^	31.86	<0.0001

Note: ^*∗∗∗*^ = very highly significant, ^*∗∗*^ = highly significant, ^*∗*^ = significant, and ^ns^ = not significant at *α* = 0.05.

**Table 2:** Effect of BAP and BAP*∗*IBA on *in vitro* shoot induction of korarima.

BAP (mg/l)	IBA (mg/l)	Induction (%) (mean + SD)	NS (mean + SD)	SH (cm) (mean ± SD)	NL (mean + SD)
0	0	56.25^e^ ± 8.54	1.68^c^ ± 0.32	3.61^b^ ± 0.76	5.85^d^ ± 0.43
0.5	0	73.12^cd^ ± 4.08	1.71^c^ ± 0.04	2.87^c^ ± 0.70	4.20^e^ ± 0.22
1	0	90.00^a^ ± 2.50	2.32^b^ ± 0.01	4.15^bc^ ± 0.70	6.58^c^ ± 0.3
1.5	0	91.25^a^ ± 2.31	2.85^ab^ ± 0.24	4.38^a^ ± 0.70	6.81^bc^ ± 0.26
2	0	78.75^c^ ± 2.50	1.69^c^ ± 0.24	3.72^b^ ± 0.46	5.39^de^ ± 0.12
2.5	0	66.25^d^ ± 5.82	1.59^c^ ± 0.38	3.08^c^ ± 0.62	4.47^e^ ± 0.22
3	0	53.12^e^ ± 2.50	1.51^c^ ± 0.32	2.62^cd^ ± 0.26	3.24^f^ ± 0.19
0.5	0.1	55.00^e^ ± 4.08	1.11^d^ ± 0.01	1.58^e^ ± 0.07	2.57^g^ ± 0.06
1	0.1	78.75^c^ ± 2.50	2.57^b^ ± 0.07	2.27^d^ ± 0.03	5.85^d^ ± 0.07
1.5	0.1	93.75^a^ ± 2.50	3.33^a^ ± 0.06	4.35^a^ ± 0.10	8.85^a^ ± 0.52
2	0.1	86.25^b^ ± 2.50	3.09^a^ ± ± 0.04	4.10^a^ ± 0.08	7.11^b^ ± 0.04
2.5	0.1	77.50^c^ ± 2.89	2.86^ab^ ± 0.07	3.61^b^ ± 0.28	5.34^de^ ± 0.03
3	0.1	73.75^cd^ ± 4.79	2.33^b^ ± 0.03	3.48^bc^ ± 0.28	4.20^e^ ± 0.06
0.5	0.2	41.25^f^ ± 2.50	0.73^e^ ± 0.02	1.21^f^ ± 0.04	1.42^i^ ± 0.03
1	0.2	66.25^d^ ± 2.50	1.83^c^ ± 0.03	2.09^de^ ± 0.03	2.39^g^ ± 0.04
1.5	0.2	91.25^a^ ± 2.50	2.07^bc^ ± 0.02	3.41^bc^ ± 0.08	5.55^d^ ± 0.18
2	0.2	73.75^cd^ ± 2.50	2.92^ab^ ± 0.03	3.06^c^ ± 0.02	4.49^e^ ± 0.05
2.5	0.2	61.25^de^ ± 2.50	2.65^b^ ± 0.04	2.50^cd^ ± 0.03	4.26^e^ ± 0.05
3	0.2	40.00^f^ ± 4.08	2.81^ab^ ± 0.02	2.38^d^ ± 0.05	2.07^h^ ± 0.04
Coefficient of variation		7.65	13.30	10.39	8.10

Means with the same letter in a column are not significantly different at *α* = 0.05. NS: number of shoots, SH: shoot height, LN: number of leaves.

**Table 3:** ANOVA summary of effect of 2,4-D, KN, and NAA, on *in vitro* callus induction of *A. corrorima*.

Source	DF	Mean square	*F* value	Pr > *F*
2,4-D	5	4606.05^*∗∗∗*^	293.64	<0.0001
NAA	3	2445.18^*∗∗∗*^	155.88	<0.0001
2,4-D*∗*KN	7	2662.73^*∗∗∗*^	169.75	<0.0001
2,4-D*∗*NAA	7	640.94^*∗∗∗*^	40.86	<0.0001
KN*∗*NAA	5	53.24^*∗∗∗*^	3.39	0.0085

Note: ^*∗∗∗*^ = very highly significant, ^*∗∗*^ = highly significant, ^*∗*^ = significant, and ^ns^ = not significant at *α* = 0.05.

**Table 4:** Effect of 2, 4-D, KN, and NAA on *in vitro* callus induction of korarima.

2,4-D (mg/l)	KN (mg/l)	NAA (mg/l)	CI % (mean ± SD)	Color	Texture
0	0	0	22.78^f^ ± 1.75	White	Friable
1	0	0	58.33^bc^ ± 3.25	White-yellow	Friable
2	0	0	38.89^de^ ± 1.33	White-yellow	Friable
3	0	0	25.00^f^ ± 1.23	White	Friable
4	0	0	16.67^g^ ± 2.89	White	Friable
5	0	0	16.67^g^ ± 2.02	White	Friable
0	0	1	41.67^d^ ± 2.89	White	Friable
0	0	2	27.25^f^ ± 2.89	White	Compact
0	0	3	25.00^f^ ± 5.00	White	Friable
1	0.5	0	8.33^h^ ± 2.88	White	Compact
1	1	0	41.67^d^ ± 1.77	White	Friable
2	0.5	0	77.50^a^ ± 3.00	White	Friable
2	1	0	33.33^bc^ ± 2.88	White-yellow	Friable
3	0.5	0	66.67^b^ ± 2.64	White-yellow	Friable
3	1	0	58.33^bc^ ± 2.89	White-yellow	Friable
4	0.5	0	33.33^e^ ± 2.89	White	Friable
4	1	0	50.00^c^ ± 3.00	White-yellow	Friable
1	0	0.5	41.67^d^ ± 2.89	White	Friable
1	0	1	75.00^a^ ± 3.00	White-yellow	Friable
2	0	0.5	25.00^f^ ± 0.00	White	Friable
2	0	1	41.67^d^ ± 2.89	White-yellow	Friable
3	0	0.5	16.67^g^ ± 2.89	White	Compact
3	0	1	33.33^e^ ± 2.89	White	Friable
4	0	0.5	8.33^h^ ± 2.89	White	Compact
4	0	1	41.67^d^ ± 2.64	White	Friable
0	1	0.5	16.67^g^ ± 2.89	White	Friable
0	1	1	33.33^e^ ± 2.89	White	Friable
0	2	0.5	8.33^h^ ± 2.88	White	Compact
0	2	1	16.67^g^ ± 2.89	White	Friable
0	3	0.5	0.00^i^ ± 0.00	None	None
0	3	1	52.78^c^ ± 4.75	White	Friable
Coefficient of variation			12.45		12.45

Means with the same letter in a column are not significantly different at *α* = 0.05. CI: callus induction.

**Table 5:** ANOVA summary of effect of BAP, TDZ, BAP*∗*IBA, and TDZ*∗*IBA on *in vitro* plantlet regeneration of *A. corrorima*.

Source	DF	Mean square	*F* value	Pr > *F*
BAP	5	737.62^*∗∗∗*^	85.11	<0.0001
TDZ	5	226.06^*∗∗∗*^	26.08	<0.0001
BAP*∗*IBA	5	487.69^*∗∗∗*^	56.27	<0.0001
TDZ*∗*IBA	5	157.48^*∗∗∗*^	18.17	<0.0001

Note: ^*∗∗∗*^ = very highly significant, ^*∗∗*^ = highly significant, ^*∗*^ = significant, and ^ns^ = not significant at *α* = 0.05.

**Table 6:** Effect of BAP, TDZ, BAP*∗*IBA, and TDZ*∗*IBA on *in vitro* regeneration of korarima.

BAP (mg/l)	TDZ (mg/l)	IBA (mg/l)	Regeneration % (mean ± SD)	NS (mean ± SD)	SH (cm) mean ± SD
0	0	0	0.00^f^ ± 0.00	0.00^e^ ± 0.00	0.00^d^ ± 0.00
0.5	0	0	25.00^c^ ± 5.00	2.33^b^ ± 0.15	1.6 0^b^ ± 0.17
1	0	0	38.33^a^ ± 2.89	3.75^a^ ± 0.13	1.8^ab^ ± 0.00
1.5	0	0	18.33^d^ ± 2.89	1.67^c^ ± 0.11	1.50^b^ ± 0.10
2	0	0	11.67^de^ ± 2.89	1.12^d^ ± 0.11	0.85^c^ ± ± 0.05
3	0	0	0.00^f^ ± 0.00	0.00^e^ ± 0.00	0.00^d^ ± 0.00
0	0.5	0	18.33^d^ ± 2.89	1.67^c^ ± 0.11	1.30^bc^ ± 0.00
0	1	0	25.00^c^ ± 5.00	2.17^b^ ± 0.21	1.90^ab^ ± 0.20
0	1.5	0	18.33^d^ ± 2.89	1.30^d^ ± 0.10	1.20^bc^ ± 0.10
0	2	0	6.67^e^ ± 2.89	1.25^d^ ± 0.05	0.90^c^ ± 0.10
0	3	0	0.00^f^ ± 0.00	0.00 ± 0.00	0.00^d^ ± 0.00
1	0	0.5	31.67^b^ ± 2.89	2.50^ab^ ± 0.20	2.10^a^ ± 0.26
1	0	1	13.30^de^ ± 2.89	1.00^d^ ± 0.00	0.78^c^ ± 0.10
2	0	0.5	43.33^a^ ± 2.89	4.34^a^ ± 0.15	2.40^a^ ± 0.00
2	0	1	13.33^de^ ± 2.89	1.12^d^ ± 0.00	0.83^c^ ± 0.25
3	0	0.5	11.67^de^ ± 2.89	1.00^d^ ± 0.00	0.80^c^ ± 0.00
3	0	1	6.67^e^ ± 2.89	0.00^e^ ± 0.00	0.00^d^ ± 0.00
0	1	0.5	16.67^d^ ± 2.89	1.50^cd^ ± 0.00	1.13^bc^ ± 0.15
0	1	1	13.33^de^ ± 2.89	1.13^d^ ± 0.10	0.90^c^ ± 0.00
0	2	0.5	45.00^a^ ± 5.00	4.45^a^ ± 0.10	1.80^b^ ± 0.10
0	2	1	13.33^de^ ± 2.89	1.00^d^ ± 0.00	0.93^c^ ± 0.15
0	3	0.5	18.33^d^ ± 2.89	1.73^c^ ± 0.06	1.30^bc^ ± 0.00
0	3	1	8.33^e^ ± 2.89	1.00^d^ ± 0.00	0.75^c^ ± 0.00
Coefficient of variation			19.20	7.05	19.20

Means with the same letter in a column are not significantly different at *α* = 0.05.; NS: number of shoots, SH: shoot height.

**Table 7:** ANOVA summary of effect of BAP, TDZ, KN, and BAP*∗*IBA on *in vitro* shoot multiplication of *A. corrorima*.

Source	DF	Mean square	*F* value	Pr > *F*
BAP	6	11.25^*∗∗∗*^	117.93	<0.0001
TDZ	6	4.04^*∗∗∗*^	42.35	<0.0001
KN	6	7.64^*∗∗∗*^	80.01	<0.0001
BAP*∗*IBA	8	6.49^*∗∗∗*^	68.04	<0.0001

Note: ^*∗∗∗*^ = very highly significant, ^*∗∗*^ = highly significant, ^*∗*^ = significant, and ^ns^ = not significant at *α* = 0.05.

**Table 8:** Effect of BAP, TDZ, KN, and BAP*∗*IBA on *in vitro* shoot multiplication of korarima.

BAP (mg/l)	TDZ (mg/l)	KN (mg/l)	IBA (mg/l)	NS (mean ± SD)	SH (mean ± SD)
0	0	0	0	5.45^f^ ± 0.15	3.28^cd^ ± 0.09
0.5	0	0	0	6.61^e^ ± 0.35	3.73^c^ ± 0.23
1	0	0	0	7.25^d^ ± 0.35	3.92^bc^ ± 0.23
1.5	0	0	0	10.83^a^ ± 0.35	5.37^a^ ± 0.23
2	0	0	0	9.22^b^ ± 0.35	4.59^ab^ ± 0.23
2.5	0	0	0	7.23^d^ ± 0.35	3.69^c^ ± 0.23
0	0.5	0	0	9.05^bc^ ± 0.35	3.46^c^ ± 0.23
0	1	0	0	9.25^b^ ± 0.35	5.21^a^ ± 0.23
0	1.5	0	0	8.21^c^ ± 0.35	4.91^a^ ± 0.23
0	2	0	0	7.18^d^ ± 0.35	4.28^b^ ± 0.23
0	2.5	0	0	7.66^d^ ± 0.35	3.11^cd^ ± 0.23
0	0	0.5	0	6.69^e^ ± 0.35	3.19^cd^ ± 0.23
0	0	1	0	7.23^d^ ± 0.35	3.62^c^ ± 0.23
0	0	1.5	0	8.20^c^ ± 0.35	4.31^b^ ± 0.23
0	0	2	0	9.66^b^ ± 0.35	4.72^ab^ ± 0.23
0	0	2.5	0	7.18^d^ ± 0.35	4.12^b^ ± 0.23
1	0	0	0.1	6.76^de^ ± 0.43	2.39^d^ ± 0.08
1	0	0	0.3	4.86^g^ ± 0.25	2.07^f^ ± 0.08
1	0	0	0.5	2.92^i^ ± 0.17	1.89^f^ ± 0.17
2	0	0	0.1	9.64^b^ ± 0.08	4.79^a^ ± 0.13
2	0	0	0.3	7.91^cd^ ± 0.17	4.89^a^ ± 0.10
2	0	0	0.5	6.28^e^ ± 0.12	3.39^cd^ ± 0.08
3	0	0	0.1	5.17^f^ ± 0.12	2.49^e^ ± 0.17
3	0	0	0.3	4.82^g^ ± 0.17	3.22^cd^ ± 0.09
3	0	0	0.5	3.94^h^ ± 0.19	2.08^f^ ± 0.06
Coefficient of variation				7.80	6.24

Means with the same letter in a column are not significantly different at *α* = 0.05. NS: number of shoots, SH: shoot height.

**Table 9:** ANOVA summary of effect of IBA, IAA, and NAA on *in vitro* rooting of *A. corrorima*.

Source	DF	Mean square	*F* value	Pr > *F*
IBA	6	39.32^*∗∗∗*^	201.54	<0.0001
IAA	6	86.15^*∗∗∗*^	441.60	<0.0001
NAA	6	11.97^*∗∗∗*^	61.37	<0.0001

Note: ^*∗∗∗*^ = very highly significant, ^*∗∗*^ = highly significant, ^*∗*^ = significant, and ^ns^ = not significant at *α* = 0.05.

**Table 10:** Effect of IBA, IAA, and NAA on *in vitro* shoot elongation and rooting of korarima.

IBA (mg/l)	IAA (mg/l)	NAA (mg/l)	NR (mean ± SD)	RL (mean ± SD)	SH (mean ± SD)
0	0	0	5.64^e^ ± 0.19	2.89^h^ ± 0.10	2.36^e^ ± 0.12
0.25	0	0	12.53^b^ ± 0.45	6.73^d^ ± 0.24	5.60^c^ ± 0.28
0.5	0	0	16.26^a^ ± 0.45	8.15^bc^ ± 0.24	6.33^b^ ± 0.28
0.75	0	0	16.59^a^ ± ± 0.45	9.16^a^ ± 0.24	7.60^a^ ± 0.28
1	0	0	14.12^ab^ ± 0.45	8.73^b^ ± 0.24	7.20^a^ ± 0.28
1.25	0	0	10.64^b^ ± 0.45	6.10^de^ ± 0.24	5.39^c^ ± 0.28
1.5	0	0	9.59^bc^ ± 0.45	5.18^f^ ± 0.24	4.02^d^ ± 0.28
0	0.25	0	15.60^a^ ± 0.45	8.70^b^ ± 0.24	6.67^b^ ± 0.28
0	0.5	0	18.59^a^ ± 0.45	9.71^a^ ± 0.24	7.32^a^ ± 0.28
0	0.75	0	16.99^a^ ± 0.45	8.84^b^ ± 0.24	6.60^b^ ± 0.28
0	1	0	13.69^ab^ ± 0.45	7.40^c^ ± 0.24	5.62^c^ ± 0.28
0	1.25	0	10.02^b^ ± 0.45	5.58^ef^ ± 0.24	4.91^cd^ ± 0.28
0	1.5	0	8.28^c^ ± 0.45	4.68^g^ ± 0.24	4.18^d^ ± 0.28
0	0	0.25	12.69^ab^ ± 0.45	7.71^c^ ± 0.24	5.83^c^ ± 0.28
0	0	0.5	13.36^ab^ ± 0.45	8.44^b^ ± 0.24	7.29^a^ ± 0.28
0	0	0.75	15.88^a^ ± 0.45	8.90^b^ ± 0.24	6.63^b^ ± 0.28
0	0	1	12.59^b^ ± 0.45	7.26^c^ ± 0.24	5.73^c^ ± 0.28
0	0	1.25	7.26^d^ ± 0.45	4.31^g^ ± 0.24	4.24^d^ ± 0.28
0	0	1.5	11.05^b^ ± 0.45	5.33^f^ ± 0.24	4.21^d^ ± 0.28
Coefficient of variation			6.56	5.41	6.19

Means with the same letter in a column are not significantly different at *α* = 0.05. NR: number of roots, RL: root length, SH: shoot height.

## Data Availability

Data will be made available on reasonable request to the corresponding author.

## Abbreviations

2, 4-D: 2, 4-Dichlorophenoxyacetic acid
ANOVA: Analysis of variance
BAP: 6-Benzylaminopurine
CRD: Completely randomized design
IAA: Indole -3-acetic acid
IBA: Indole-3-butric acid
JARC: Jimma Agricultural Research Center
KN: Kinetin
MS: Murashige and Skoog
NAA: *α*-Naphthalene acetic acid
NaOCl: Sodium hypochlorite
PGR: Plant growth regulator
REGWQ: Ryan–Einot–Gabriel-–Welsch range test (Q)
SAS: Statistical analysis system
TDZ: Thidiazuron.
